# Instrumentos que avaliam a mobilidade de crianças e adolescentes com transtorno do espectro autista: Uma revisão sistemática e mapa de decisão

**DOI:** 10.1111/dmcn.70144

**Published:** 2025-12-29

**Authors:** Arthur Felipe Barroso de Lima, Amanda Cristina Fernandes, Amanda Alves Rodrigues Soares, Hércules Ribeiro Leite, Ricardo Rodrigues de Sousa Junior

**Affiliations:** ^1^ Graduação em Fisioterapia Universidade Federal de Minas Gerais Belo Horizonte Brasil; ^2^ Programa de Pós‐Graduação em Ciências da Reabilitação, Escola de Educação Física, Fisioterapia e Terapia Ocupacional Universidade Federal de Minas Gerais Belo Horizonte Brasil

## Abstract

**Objetivos:**

Identificar os instrumentos padronizados usados para avaliar aspectos de mobilidade em crianças e adolescentes com transtorno do espectro autista (TEA), analisar a qualidade de suas propriedades psicométricas e seu nível de evidência, e desenvolver um mapa de decisão clínica para esses instrumentos.

**Métodos:**

Os artigos foram selecionados e as características dos estudos foram extraídas. A qualidade metodológica dos estudos selecionados foi analisada usando o checklist de Risco de Viés COSMIN. A qualidade da evidência para cada propriedade de medida foi definida usando uma versão modificada do sistema GRADE (Grading of Recommendations Assessment, Development and Evaluation).

**Resultados:**

Onze instrumentos foram analisados em 11 estudos. Desses instrumentos, três são direcionados para avaliação de desempenho e oito para avaliação de capacidade. Os estudos selecionados avaliaram as propriedades psicométricas das Escalas de Comportamento Adaptativo Vineland, Avaliação Motora Grossa de Crianças e Adolescentes com Transtorno do Espectro Autista, Ignite Challenge, Inventário de Avaliação de Incapacidade Pediátrica com Teste Adaptativo por Computador, Escalas Miller de Função e Participação, Escalas de Desenvolvimento Motor Peabody, Segunda Edição, Teste de Desenvolvimento Motor Grosso, Segunda e Terceira Edições, Timed Up and Go, Questionário de Transtorno de Coordenação do Desenvolvimento e a Bateria de Avaliação do Movimento para Crianças, Segunda Edição. Desses instrumentos, nove foram desenvolvidos para a avaliação de crianças com desenvolvimento típico e crianças com deficiências no geral, e foram validados para a população com TEA (81,8%). Os outros dois instrumentos (18,2%) foram especificamente desenvolvidos para a avaliam o público com TEA.

**Interpretação:**

A maioria (56,51%) das propriedades de medida dos instrumentos apresentou evidência baixa ou muito baixa devido ao risco de viés e imprecisão, reforçando a importância de mais estudos para fortalecer a validade e a aplicabilidade dessas avaliações.

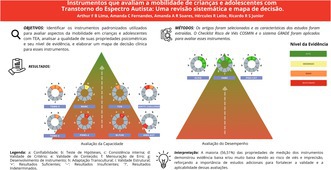

**O que este artigo acrescenta**

- Ainda faltam estudos robustos que confirmem o nível de evidência desses instrumentos para o transtorno do espectro autista (TEA).
- Um mapa de decisão baseado nas evidências orienta a seleção de avaliações apropriadas dos aspectos de mobilidade no TEA.
- São necessárias análises futuras das propriedades de medição dos instrumentos.

**SIGLAS**
CIF: Classificação de Funcionalidade, Incapacidade e Saúde
DCDQ: Questionário de Transtorno do Desenvolvimento da Coordenação
GMA‐AUT: Avaliação Motora Grossa de Crianças e Adolescentes com Transtorno do Espectro Autista
MABC‐2: Bateria de Avaliação do Movimento para Crianças – segunda versão
M‐FUN: Escalas de Função e Participação de Miller
PDMS‐2: Escalas de Desenvolvimento Motor de Peabody, Segunda Edição
PEDI‐CAT: Inventário de Avaliação Pediátrica de Incapacidade ‐ Teste adaptativo por computador
TEA: Transtorno do Espectro do Autismo
TGMD‐2: Teste de Desenvolvimento Motor Grosso ‐ Segunda edição
TGMD‐3: Teste de Desenvolvimento Motor Grosso ‐ Segunda edição
TUG: Timed Up and Go
VABS: Escalas de Comportamento Adaptativo de Vineland

O Transtorno do Espectro Autista (TEA) é caracterizado principalmente por déficits na interação social, na comunicação e pela presença de comportamentos, interesses ou atividades restritos e repetitivos.^1^ Nos últimos anos, a prevalência do TEA aumentou significativamente. Dados recentes indicam que uma em cada 36 crianças nos Estados Unidos foi diagnosticada com TEA.^2^ As causas da condição são multifatoriais, envolvendo fatores ambientais e genéticos, o que contribui para a variabilidade na apresentação clínica e no funcionamento desses indivíduos.^3^

Conforme a Classificação Internacional de Funcionalidade, Incapacidade e Saúde (CIF), a funcionalidade engloba as funções e estruturas do corpo, as atividades, a participação, bem como os fatores contextuais (pessoais e ambientais).^4^ Indivíduos com TEA podem exibir vários comprometimentos que dificultam sua funcionalidade. Esses comprometimentos frequentemente resultam em limitações em diferentes áreas da vida, especialmente na mobilidade, que tende a ser subavaliada nessa população.^5^, ^6^ Segundo a CIF, a mobilidade é um aspecto motor fundamental dentro do domínio de atividade da CIF, envolvendo a capacidade de se mover no ambiente (por exemplo, mudar e manter posições corporais), transportar ou mover objetos (por exemplo, carregar ou manusear) e a locomoção (por exemplo, andar e se deslocar).^4^ Estudos recentes evidenciam uma alta prevalência de limitação nesses aspectos de mobilidade em crianças e adolescentes com TEA.^6^, ^7^, ^8^, ^9^

Evidências relacionadas ao TEA destacam limitações de mobilidade em habilidades motoras finas, de equilíbrio e coordenação, locomotoras e de controle de objetos.^6^ Esses aspectos podem ocorrer devido a déficits na práxis motora, hipotonia e baixa resistência encontrados nesse grupo de crianças e adolescentes.^6^, ^7^, ^8^, ^9^ Essas limitações, em conjunto com as limitações sociais, podem dificultar a participação de crianças e adolescentes em atividades físicas, uma vez que eles apresentam níveis mais baixos de atividade física em comparação com seus pares com desenvolvimento típico.^10^, ^11^ Além disso, as limitações de mobilidade podem atuar como barreiras para a plena participação em outras áreas da vida de crianças e adolescentes com TEA, impactando sua autonomia e integração social e familiar.^12^, ^13^, ^14^

Instrumentos padronizados para avaliar a interação social, a comunicação e os comportamentos restritos e repetitivos no TEA são bem estabelecidos; no entanto, as ferramentas para avaliar a mobilidade nesta população permanecem limitadas.^15^, ^16^ Existem vários instrumentos para avaliar a capacidade (execução de tarefas em um ambiente padronizado) e o desempenho (execução de tarefas no ambiente cotidiano da criança) da mobilidade em crianças com transtornos do neurodesenvolvimento.^4^ Capacidade e desempenho são aspectos distintos, mas complementares, da mobilidade em crianças com TEA — por exemplo, a capacidade reflete a precisão com que uma criança executa uma tarefa motora, enquanto o desempenho reflete se é necessário auxílio para concluir a tarefa motora na vida diária.

A aplicação dessas ferramentas a crianças e adolescentes com TEA, no entanto, pode ser complexa. Alguns testes podem não capturar dificuldades motoras relevantes.^17^ Além disso, interesses restritos, atenção limitada e dificuldades de envolvimento com o avaliador podem reduzir a colaboração do paciente no teste, enquanto comprometimentos cognitivos naqueles que requerem níveis mais altos de suporte podem dificultar a compreensão das instruções. Ademais, muitos especialistas em movimento, como fisioterapeutas, relatam falta de experiência, confiança e treinamento formal ao avaliar a mobilidade nessa população.^18^ Uma compreensão mais sólida das avaliações de mobilidade padronizadas pode melhorar as habilidades clínicas dos terapeutas e ajudá‐los a enfrentar esses desafios com mais eficácia.

Recentemente, revisões sistemáticas investigaram ferramentas gerais de avaliação motora em crianças e adolescentes com TEA.^17^, ^19^ Esses estudos apresentaram instrumentos principalmente para crianças com alto risco de autismo, sem incluir adolescentes e com falta de foco nos aspectos de mobilidade. Além disso, a qualidade das propriedades de medida dessas ferramentas não foi avaliada.^17^, ^19^ Isso pode representar uma limitação para os clínicos na seleção dos instrumentos mais apropriados com base em seu construto e no nível de evidência de suas propriedades de medida.

Portanto, analisar as ferramentas disponíveis sobre a avaliação dos aspectos de mobilidade e suas propriedades de medida pode fornecer recomendações baseadas em evidências, auxiliando os clínicos na seleção de escalas de avaliação padronizadas e adequadas para avaliar crianças e adolescentes com TEA. Consequentemente, o presente estudo visa identificar as ferramentas padronizadas utilizadas para avaliar aspectos de mobilidade em crianças e adolescentes com TEA. Adicionalmente, analisará a qualidade de suas propriedades psicométricas e seu nível de evidência, e desenvolver um mapa de decisão clínica para facilitar o raciocínio clínico durante o processo de avaliação dessa população.

## MÉTODOS

Esta revisão sistemática foi conduzida com base nos critérios do Preferred Reporting Items for Systematic Reviews and Meta‐Analyses — Consensus‐based Standards for the selection of health Measurement Instruments (PRISMA‐COSMIN) para Instrumentos de Medição de Resultados em Saúde e seguiu as diretrizes COSMIN para a realização de revisões sistemáticas de medidas de resultado.^20^, ^21^ Esta revisão foi registrada no International Prospective Register of Systematic Reviews (PROSPERO) sob o número de registro CRD42023485879.

### Estratégia de busca e seleção

Uma busca foi realizada nas bases de dados PubMed, Web of Science, Embase, PsycInfo, Scopus e SciELO em outubro de 2023. A busca foi atualizada em agosto de 2025 para garantir a captura de novos estudos. O processo de seleção foi executado por dois revisores independentes (AFBL e AARS). A estratégia de busca foi desenvolvida usando palavras‐chave relacionadas à estrutura PICO. Seguindo a estrutura PICO (População, Indicador, Comparação e Resultado), este estudo respondeu à pergunta: “Entre crianças e adolescentes com TEA (P), quais ferramentas padronizadas (I) são usadas para avaliar aspectos de mobilidade, e qual é a qualidade de suas propriedades psicométricas e nível de evidência para esta população específica (O)?” O material suplementar 1 apresenta as palavras‐chave para a busca deste estudo, que foi baseada no filtro de busca COSMIN para revisão sistemática de instrumentos.^22^

As listas de referência dos estudos selecionados e de revisões anteriores sobre instrumentos para crianças com TEA^17^, ^19^ foram pesquisadas manualmente para garantir que todos os estudos potenciais que avaliassem propriedades de medida de avaliações de mobilidade em crianças e adolescentes com TEA fossem incluídos. Adicionalmente, foram conduzidas buscas complementares usando os nomes das avaliações encontradas em nossa busca principal e aquelas mencionadas em estudos de revisão anteriores. Para essas buscas adicionais, incluímos como palavras‐chave o nome do instrumento juntamente com o filtro de busca COSMIN.^22^

### Critérios de Elegibilidade

Este estudo incluiu estudos psicométricos que investigaram uma ou mais propriedades de medida de instrumentos de avaliação padronizados, como confiabilidade e consistência interna, desenvolvidos para avaliar aspectos da mobilidade em crianças e adolescentes com TEA. A mobilidade foi considerada conforme definida na CIF no componente Atividade (ou seja, mudar e manter a posição do corpo; carregar, mover e manusear objetos; andar e se mover; e locomover‐se utilizando transporte).4 Portanto, instrumentos que avaliavam outros componentes foram considerados se fossem primariamente direcionados a aspectos de mobilidade (>60% dos itens do instrumento). Esses aspectos incluem locomoção, transferências, manipulação de objetos e outros, para indivíduos com TEA na faixa etária de 0 a 21 anos. Os estudos deveriam incluir crianças e adolescentes diagnosticados com TEA em mais de 50% de sua amostra. Isso também incluiu crianças com outros diagnósticos de neurodesenvolvimento, como síndromes genéticas, desde que houvesse informação sobre comorbidade de TEA ou sintomas centrais do autismo na amostra do estudo.

Não houve restrições quanto ao idioma ou ano de publicação. Foram excluídos os seguintes estudos: (a) aqueles que não incluíam amostras predominantemente compostas por crianças e adolescentes diagnosticados com TEA (<50%), (b) resumos e (c) instrumentos com foco em desfechos não relacionados a aspectos de mobilidade.

### Extração dos dados e análise

Dois revisores independentes (AFBL e AARS) removeram as duplicatas e triaram os títulos e resumos. Subsequentemente, os textos completos dos artigos potencialmente elegíveis foram avaliados. Divergências entre os revisores foram resolvidas por um terceiro revisor (ACF). Informações sobre as características dos estudos e instrumentos (como os desfechos avaliados e o tempo de aplicação) e da população (como tamanho da amostra, idade e sexo), país onde o estudo foi conduzido, propriedades de medida avaliadas e resultados (ou seja, índices psicométricos) foram extraídos.

Conforme as diretrizes COSMIN para a realização de revisões sistemáticas de instrumentos, foram extraídos e investigados dados das seguintes propriedades de medida: (1) validade de conteúdo, (2) validade estrutural (incluindo a estrutura fatorial), (3) consistência interna, (4) validade transcultural e medida de invariância, (5) confiabilidade, (6) medida de erro, (7) validade de critério, (8) teste de hipóteses para validade de construto, (9) responsividade, e (10) desenvolvimento do instrumento. Definições operacionais de cada uma dessas propriedades de medida, bem como informações sobre seus critérios de qualidade, podem ser encontradas nas diretrizes COSMIN.^20^

Também extraímos informações sobre o tipo de instrumento conforme os descritores do domínio de Atividade da CIF: desempenho e capacidade. Desempenho é definido como a execução de uma tarefa no ambiente habitual, e capacidade é a execução de uma tarefa em um ambiente padronizado.^4^ Na reabilitação pediátrica, o desempenho é comumente avaliado por questionários respondidos pelos pais sobre a realização das atividades pelos seus filhos em seu próprio ambiente. A capacidade é comumente avaliada por avaliações motoras objetivas padronizadas.

### Qualidade metodológica dos estudos

#### Risco de Viés

A qualidade metodológica dos estudos selecionados foi avaliada usando o Checklist COSMIN para Risco de Viés (Risk of Bias Checklist).^20^, ^23^, ^24^ Cada propriedade de medida avaliada em um estudo é classificada em uma escala de 4 pontos: muito boa, adequada, duvidosa ou inadequada. A qualidade metodológica geral para cada propriedade é determinada pela classificação mais baixa entre os critérios relevantes (ou seja, o princípio do “pior resultado conta”). O Checklist COSMIN para Risco de Viés inclui critérios distintos para diferentes propriedades de medida; portanto, um único estudo pode receber classificações diferentes para cada propriedade avaliada.^20^, ^23^, ^24^

#### Qualidade das propriedades de medida

As propriedades de medida relatadas nos estudos foram avaliadas por meio dos critérios de qualidade COSMIN.^25^ Cada propriedade identificada nos estudos selecionados foi classificada em três categorias, a saber: “(+) suficiente”, quando os índices psicométricos estatísticos atendiam aos critérios COSMIN; “(−) insuficiente”, quando não atendiam a esses critérios; ou “(?) indeterminada”, nos casos em que as informações disponíveis sobre os índices psicométricos eram insuficientes.

O risco de viés e a qualidade das propriedades de medida foram avaliados de forma independente por dois revisores investigadores (AFBL e AARS) e, caso houvesse discrepância, um terceiro revisor foi responsável pela definição (ACF).

### Síntese dos resultados

Os dados de todos os estudos que avaliaram o mesmo instrumento e as mesmas propriedades de medida foram sintetizados para determinar sua qualidade metodológica geral e os critérios gerais de qualidade para as propriedades de medida, conforme recomendado por Mokkink et al.^20^ A qualidade metodológica geral do instrumento foi estabelecida somando o número de estudos classificados como “muito bom”, “adequado”, “duvidoso” e “inadequado”. A avaliação geral de cada propriedade de medida dos instrumentos foi classificada em quatro categorias: (+) Suficiente, quando a maioria das propriedades (mais de 50%) foi classificada como “+”; (−) Insuficiente, quando a maioria (mais de 50%) foi classificada como “–”; (?) Indeterminada, quando a maioria das propriedades (mais de 50%) foi classificada como “?”; e (±) Inconsistente, quando os resultados apresentaram discrepâncias notáveis, como uma distribuição equilibrada (por exemplo, 50% “+” e 50% “–”).

### Classificação da qualidade das evidências

A qualidade da evidência para cada propriedade de medida foi definida utilizando uma versão modificada do sistema GRADE proposta por Mokkink e colaboradores.^20^ O nível de evidência é classificado como “alto”, “moderado”, “baixo” ou “muito baixo”. Neste sistema, quatro fatores são considerados para classificar a qualidade da evidência para as propriedades psicométricas de um instrumento: risco de viés, inconsistência, imprecisão e indireção. De modo geral, a abordagem GRADE começa com um nível de evidência de alta qualidade, que pode ser rebaixado gradualmente para moderado, baixo ou muito baixo, dependendo do número e da gravidade das limitações identificadas nesses quatro domínios.

O primeiro fator, risco de viés, refere‐se à qualidade metodológica dos estudos incluídos. O nível de rebaixamento depende da gravidade dos problemas metodológicos. A evidência é rebaixada em um nível (risco de viés sério) quando os estudos são de qualidade “duvidosa” ou apenas um estudo de qualidade “adequada” está disponível; em dois níveis (risco de viés muito sério) quando múltiplos estudos mostram qualidade “inadequada” na avaliação de risco de viés ou apenas um estudo de qualidade “duvidoso” existe; e em três níveis (risco de viés extremamente sério) quando apenas um estudo “inadequado” está disponível.^20^

O segundo fator, inconsistência, diz respeito à variabilidade nos resultados dos estudos para a mesma propriedade de medida. Se os resultados entre os estudos forem inconsistentes (±) na qualidade das propriedades de medida, e nenhuma explicação razoável puder ser fornecida (por exemplo, devido a diferenças nas populações ou versões do instrumento), a evidência deve ser rebaixada. É importante notar que quando as propriedades de medida são classificadas como indeterminadas (?) na avaliação de qualidade, seu nível de evidência não é classificado.

O terceiro fator, imprecisão, conforme os padrões COSMIN, relaciona‐se ao tamanho total da amostra na qual a avaliação geral se baseia. Tamanhos de amostra pequenos reduzem a confiança na estabilidade dos achados. A evidência é rebaixada em um nível (imprecisão séria) quando o tamanho total da amostra é inferior a 100 participantes, e em dois níveis (imprecisão muito séria) quando é inferior a 50.^20^

Finalmente, a evidência indireta reflete o grau em que a evidência é diretamente aplicável à população, construto ou contexto de interesse. A evidência é considerada indireta quando os estudos incluídos foram conduzidos em uma população diferente (por exemplo, populações diferentes do TEA), quando o instrumento foi usado em um contexto ou propósito diferente do pretendido, ou quando avaliou um construto diferente.^20^

A qualidade da evidência é interpretada da seguinte forma: “evidência alta”: podemos assumir com confiança que os resultados das propriedades psicométricas do instrumento são confiáveis; “evidência moderada”: podemos assumir moderadamente que os resultados das propriedades psicométricas do instrumento são confiáveis; “evidência baixa”: nossa suposição de confiabilidade é limitada e os resultados das propriedades psicométricas do instrumento podem diferir do que é apresentado; e “evidência muito baixa”: não podemos assumir que os resultados são confiáveis, e é provável que os resultados das propriedades psicométricas do instrumento difiram do que é apresentado, sendo inconsistentes.^20^, ^23^

A classificação final foi determinada por consenso entre dois revisores, usando um terceiro em caso de discordância. Esses procedimentos de classificação (risco de viés, qualidade das propriedades de medida, síntese dos achados e qualidade da evidência) seguiram estritamente as diretrizes COSMIN e o processo passo a passo exemplificado por Andrade e colaboradores.^20^, ^25^

## RESULTADOS

Das buscas realizadas, foram identificados 6.098 estudos, dos quais onze se mostraram elegíveis. O fluxograma com os detalhes do processo de busca e seleção está disponível no Material Suplementar 2. A maioria dos estudos relatou ter incluído crianças e adolescentes com diagnóstico confirmado de TEA. No entanto, os métodos considerados para o diagnóstico, as classificações funcionais e o nível de suporte necessário foram pouco relatados nos estudos selecionados.

Os estudos selecionados avaliaram as propriedades psicométricas de onze instrumentos, a saber: Escalas de Comportamento Adaptativo de Vineland (VABS)^26^, ^27^; Avaliação Motora Grossa de Crianças e Adolescentes com Transtorno do Espectro Autista (GMA‐AUT)^28^; Ignite Challenge^29^; Inventário de Avaliação Pediátrica de Incapacidade (PEDI‐CAT)^30^; Escalas de Função e Participação de Miller (M‐FUN)^31^; Escalas de Desenvolvimento Motor de Peabody ‐ Segunda Edição (PDMS‐2)^31^; Teste de Desenvolvimento Motor Grosso ‐ segunda e terceira edições (TGMD‐2 e 3)^32^, ^33^; Timed Up and Go (TUG)^34^; Questionário de Transtorno do Desenvolvimento da Coordenação (DCDQ)^35^; e Bateria de Avaliação do Movimento para Crianças‐2 (MABC‐2)^36^. Esses estudos foram conduzidos em sete países: Austrália (n = 1)^30^, Bélgica (n = 1)^35^, Brasil (n = 2)^28^, ^36^, Canadá (n = 1)^29^, China (n = 1)^26^, Espanha (n = 1)^34^ e Estados Unidos (n = 4)^27^, ^31^, ^32^, ^33^.

### Características dos instrumentos

A Tabela 1 apresenta as principais características e informações relativas aos instrumentos. Dos instrumentos selecionados, nove foram desenvolvidos para a avaliação de crianças típicas e validados para a população com TEA (81,8%). Os outros dois instrumentos (18,2%) foram desenvolvidos especificamente para a avaliação do público com TEA, como o GMA‐AUT e o Ignite Challenge. Dos instrumentos selecionados, três são direcionados para a avaliação de desempenho e oito para a avaliação de capacidade. Entre os instrumentos de capacidade, houve uma variabilidade de aspectos de mobilidade avaliados, como a qualidade de execução de habilidades locomotoras e de controle de objetos (TGMD‐2 e 3), a capacidade de execução de habilidades motoras básicas (GMA‐AUT), a precisão e o tempo de execução de habilidades motoras avançadas (Ignite Challenge), e as habilidades de destreza manual e equilíbrio (M‐ABC). Além disso, houve uma variabilidade na população‐alvo desses instrumentos, variando de 0 a 21 anos de idade.

**Tabela 1:** Características dos instrumentos.

Tipo de Instrumento	Instrumento	Tempo de Aplicação	Quais profissionais podem administrar essa ferramenta?	Idade	Específico para o TEA?	Certificação e materiais necessários	Desfechos
Avaliação do Desempenho	**PEDI‐CAT**	Até 60 minutos	Fisioterapeutas, terapeutas ocupacionais e profissionais interessados em avaliar o desempenho funcional	0–21 anos	Sim	Não é necessário certificado formal; a compra do formulário necessário	Avaliação do desempenho funcional em atividades de mobilidade
	**DCDQ**	20–30 min.	Fisioterapeutas e Terapeutas ocupacionais	5–15 anos.	Não	Não é necessário certificado formal; a compra do formulário necessário	Presença de Transtorno do Desenvolvimento da Coordenação
	**VABS**	Até 60 minutos	Terapeutas Ocupacionais, Psicólogos, Fonoaudiólogos	0 a 90 anos	Não	Não é necessário certificado formal; a compra do formulário necessário	Avaliação do desempenho funcional em atividades de mobilidade
Avaliação da Capacidade	**GMA‐AUT**	60 minutos	Fisioterapeutas e Terapeutas ocupacionais	4–18 anos	Sim	Não é necessário certificado formal; a compra do formulário necessário	Avaliação observacional da função motora grossa.
	**Ignite Challenge**	30–45 minutos	Fisioterapeutas e Terapeutas ocupacionais	6–14 anos	Sim	É necessário ter certificação formal	Avaliação observacional de habilidades motoras avançadas
	**M‐FUN**	45–60 minutos	Fisioterapeutas, terapeutas ocupacionais e profissionais interessados em avaliar habilidades motoras	2–7 anos	Não	Não é necessário certificado formal; a compra do formulário necessário	Avaliação observacional da função motora grossa.
	**TGMD‐2**	40–45 minutos	Fisioterapeutas, terapeutas ocupacionais e profissionais interessados em avaliar o desenvolvimento motor grosso	3–10 anos	Não	Não é necessário certificado formal; a compra do formulário necessário	Avaliação observacional de habilidades motoras fundamentais
	**TGMD‐3**	40–45 minutos	Fisioterapeutas, terapeutas ocupacionais e profissionais interessados em avaliar o desenvolvimento motor grosso	3–10 anos	Não	Não é necessário certificado formal; a compra do formulário necessário	Avaliação observacional de habilidades motoras fundamentais
	**PDMS‐2**	60 minutos	Fisioterapeutas e Terapeutas ocupacionais	0–6 anos	Não	Não é necessário certificado formal; a compra do formulário necessário	Avaliação observacional da função motora grossa.
	**TUG**	5 minutos	Fisioterapeutas, terapeutas ocupacionais e profissionais interessados em avaliar aspectos da mobilidade	Qualquer idade	Não	Não é necessário certificado formal; a compra do formulário necessário	Avaliação cronometrada da mobilidade (capacidade de ficar em pé, caminhar 3 metros, contornar um cone e sentar‐se novamente).
	**MABC‐2**	20 a 40 minutos	Fisioterapeutas, terapeutas ocupacionais e profissionais interessados em avaliar o desempenho funcional	3 to 16 anos	Não	Não é necessário certificado formal; a compra do formulário necessário	Avaliação observacional da função motora grossa e fina.

Legenda: ASD, autism spectrum disorder; DCDQ, Developmental Coordination Questionnaire; GMA‐AUT, Gross Motor Assessment of Children and Adolescents with ASD; MABC‐2, Movement Assessment Battery for Children, Second Edition; M‐FUN, Miller Function and Participation Scales; PDMS‐2, Peabody Developmental Motor Scales, Second Edition; PEDI‐CAT, Pediatric Evaluation of Disability Inventory Computer Adaptive Test for Autism; TGMD‐2, Test of Gross Motor Development, Second Edition; TGMD‐3, Test of Gross Motor Development, Third Edition; TUG, Timed Up and Go; VABS, Vineland Adaptive Behavior Scales.

### Características dos estudos e Risco de Viés

A Tabela 2 resume as principais características dos estudos. Considerando as propriedades psicométricas avaliadas, os estudos investigaram: teste de hipóteses (41,63%) (ou seja, validade concorrente e validade discriminativa); confiabilidade (teste‐reteste) (16,66%); consistência interna (12,5%); erro de medida (8,33%); validade de critério (4,16%); validade de conteúdo (4,16%); desenvolvimento do instrumento (4,16%); adaptação transcultural (4,16%); e validade estrutural (4,16%). Cinquenta por cento das propriedades psicométricas avaliadas em todos os estudos incluídos foram consideradas “muito boa” conforme o Checklist COSMIN para Risco de Viés, 29,16% como “adequadas” e 20,83% como “inadequadas”. Nenhuma propriedade foi classificada como “duvidosa”.

**Tabela 2:** Características dos estudos.

Estudo	Instrumento	País	População (Idade, Classificações funcionais)	Tamanho amostral	Propriedades de medidas avaliadas	Risco de Viés	Resultados	Critérios de Qualidade para Propriedades de Medida
*Assessing the Validity and Reliability of the Chinese \Vineland Adaptive Behavior Scales for Children With Autism Spectrum Disorder Aged 1–6* ^26^	Escalas de Comportamento Adaptativo de Vineland (VABS)	China	1–6 anos (classificações funcionais não reportadas)	2118 (230 para o Teste de Hipóteses)	Consistência interna; Validade estrutural; Testes de hipóteses (Validade Convergente).	**Consistência interna:** Muito bom **Validade estrutural:** Muito bom **Teste de hipóteses:** Muito bom	**Consistência Interna:** α = 0.93–0.99 **Validade Estrutural:** CFI = 0.90–0.99 **Teste de Hipóteses (Validade Convergente):** Correlações significativas positivas com as Escalas de Desenvolvimento de Gessel (r = 0,30‐0,60, p < 0,05)	**Consistência Interna:** Suficiente (+) **Validade Estrutural:** Suficiente (+) **Teste de Hipóteses (Validade Convergente):** Suficiente (+)
*Validating motor delays across the developmental coordination* *disorder‐questionnaire and the Vineland adaptive behavior scales* *(VABS) in children with autism spectrum disorder ASD: A SPARK* *dataset analysis* ^27^	Escalas de Comportamento Adaptativo de Vineland (VABS) e Questionário de Transtorno do Desenvolvimento da Coordenação (DCDQ)	EUA	1–10 anos (classificações funcionais não reportadas)	2644	Testes de hipóteses (Validade Concorrente).	**Teste de hipóteses:** Adequado	**Teste de Hipóteses (Validade Concorrente):** Forte correlação entre os escores VABS e os escores DCDQ (Pearson = 0,62)	**Teste de Hipóteses (Validade Concorrente):** Suficiente (+)
*Content validity of an instrument for motor assessment of youth with autism* ^28^	Avaliação Motora Grossa de Crianças e Adolescentes com Transtorno do Espectro Autista (GMA‐AUT	Brasil	Não aplicável	9	Validade de Conteúdo. Desenvolvimento de Instrumento.	**Validade de Conteúdo:** Inadequada **Desenvolvimento do Instrumento:** Inadequado	**Validade de Conteúdo:** Indice de Validade de Conteúdo: 0.88–1.00;	**Validade de Conteúdo:** Indeterminado (?) **Desenvolvimento de Instrumento:** Insuficiente (−)
*Getting into the game: evaluation of the reliability, validity, and utility of the Ignite Challenge scale for school‐aged children with autism spectrum disorder* ^29^	Ignite Challenge e Inventário de Avaliação Pediátrica de Incapacidade ‐ Teste adaptativo por computador (PEDI‐CAT)	Canadá	6 a 17 anos; ACSF‐SC níveis I e II	47	Confiabilidade; Mensuração de erro; Testes de hipóteses (Validade Discriminante e Concorrente).	**Confiabilidade:** Adequada **Mensuração de Erro:** Adequado **Teste de hipóteses:** Muito bom	**Confiabilidade:** Inter‐examinador: ICC = 0.96 Intra‐examinador: ICC = 0.91 **Mensuração de Erro:** MDC = 9.28 **Teste de Hipóteses (Validade Concorrente):** Correlações significativas entre os escores do Ignite Challenge e o domínio de mobilidade do PEDI‐CAT (r = 0,54, p < 0,0001), e Social/Cognitivo (r = 0,57, p < 0,0001) **Teste de Hipóteses (Validade Discriminante):** Ignite Challenge mostrou resultados diferentes entre faixas etárias (melhor pontuação no teste 59,4 (dp = 15,5) versus pontuação da criança mais velha 80,3 (dp = 10,1), p < 0,001), e também entre os níveis ACSF:SC (Nível I melhor pontuação 73,8 (dp = 12,8) versus Nível II pontuação 58,3 (dp = 20,9), p = 0,007).	**Confiabilidade:** Suficiente (+) **Mensuração de Erro:** Indeterminado (?) **Teste de Hipóteses (Validade Discriminante e Concorrente)** Suficiente (+)
*Reliability, Validity and Acceptability of the PEDI‐CAT with ASD Scales for Australian Children and Youth on the Autism Spectrum* ^30^	Inventário de Avaliação Pediátrica de Incapacidade ‐ Teste adaptativo por computador (PEDI‐CAT) e Escalas de Comportamento Adaptativo de Vineland (VABS)	Austrália	3–18 anos; ACSF‐SC e todos os níveis de suporte incluídos	134	Consistência interna; Confiabilidade; Teste de Hipóteses (Validade Convergente).	**Consistência interna:** Muito bom **Confiabilidade:** Adequada **Teste de hipóteses:** Muito bom	**Consistência Interna:** McDonald's Omega = 0.89–0.93 **Confiabilidade Teste‐reteste**: ICC: 0.89–0.92 **Teste de Hipóteses (Validade Convergente):** Correlações positivas significativas VABS (r = 0,51‐0,74, p < 0,05)	**Consistência Interna:** Indeterminado (?) **Confiabilidade:** Suficiente (+) **Teste de Hipóteses (Validade Convergente):** Suficiente (+)
*Concurrent Validity of Two Standardized Measures of Gross Motor Function in Young Children with Autism Spectrum Disorder* ^31^	Escalas de Função e Participação de Miller (M‐FUN) e Escalas de Desenvolvimento Motor de Peabody, Segunda Edição (PDMS‐2)	EUA	4–5 anos CARS moderado 64%) e grave (36%)	22	Teste de Hipóteses.	**Teste de hipóteses:** Adequado	**Teste de Hipóteses: (Validade Concorrente):** Correlação significativa entre a escala M‐FUN e os escores motores grosseiros do PDMS‐2 (r = 0,84, p < 0,05). **Teste de Hipóteses: (Validade Discriminante):** Forte correlação na identificação de crianças com habilidades motoras médias e atrasadas (k de Cohen = 0,77 e p < 0,05)	**Teste de Hipóteses: (Validade concorrente e discriminante):** Suficiente (+)
*The Effect of Visual Supports on Performance of the TGMD‐2 for Children with Autism Spectrum Disorder* ^32^	Teste de Desenvolvimento Motor Grosso ‐ Segunda edição (TGMD‐2)	EUA	3–10 anos (classificações funcionais não reportadas)	22	Teste de Hipóteses (Validade concorrente).	**Teste de hipóteses:** Muito bom	**Teste de Hipóteses (Validade Concorrente):** Diferença significativa entre os grupos com e sem suporte visual, p = 0,003	**Teste de Hipóteses:** Suficiente (+)
*Test of Gross Motor Development‐3 (TGMD‐3) with the Use of Visual Supports for Children with Autism Spectrum Disorder: Validity and Reliability* ^33^	Teste de Desenvolvimento Motor Grosso ‐ Terceira edição (TGMD‐3)	EUA	4–10 anos (classificações funcionais não reportadas)	14	Consistência Interna; Confiabilidade; Teste de Hipóteses (Validade Concorrente).	**Consistência Interna:** Muito bom **Confiabilidade:** Muito bom **Teste de hipóteses:** Muito bom	**Consistência Interna:** Grupo TEA: α: 0.88 Locomoção: 0.82 Habilidades com bolas: 0.5 TEA + suporte visual: α: 0.93 Locomoção: 0.93 Habilidades com bolas: 0.81 **Confiabilidade:** Grupo TEA: Teste‐reteste: ICC = 0.91 Inter‐examinador: ICC = 0.98 Intra‐examinador: ICC = 0.99 TEA + suporte visual: Teste‐reteste: ICC = 0.92 Inter‐examinador: ICC = 0.99 Intra‐examinador: ICC = 0.99 **Teste de Hipóteses (Validade concorrente):** Os escores brutos do TGMD‐3 de crianças com TEA melhoraram significativamente com o uso do suporte visual do TGMD‐3. (p = 0,01) Melhora significativa com o uso de suportes visuais (p = 0,01).	**Consistência Interna com suporte visual:** Suficiente (+) **Consistência Interna sem suporte visual:** Suficiente (+) **Confiabilidade com suporte visual:** Suficiente (+) **Confiabilidade sem suporte visual:** Suficiente (+) **Teste de Hipóteses (Validade concorrente):** Suficiente (+)
*Reliability and agreement of the timed up and go test in children and teenagers with autism spectrum disorder* ^34^	Timed Up and Go	Espanha	6–18 anos (classificações funcionais não reportadas)	50	Confiabilidade; Mensuração de Erro.	**Confiabilidade** Adequada **Mensuração de Erro:** Adequada	**Confiabilidade:** Inter‐examinador: ICC = 0.99 Intra‐examinador: ICC = 0.88 **Mensuração de Erro:** MDC: 0.06	**Confiabilidade:** Suficiente (+) **Mensuração de Erro:** Indeterminado (?)
*Evaluation of the Developmental Coordination Questionnaire (DCDQ) as a Screening Instrument for Co‐occurring Motor Problems in Children with Autism Spectrum Disorder* ^35^	Questionário de Transtorno do Desenvolvimento da Coordenação (DCDQ) e Bateria de Avaliação do Movimento para Crianças‐2 (MABC‐2)	Bélgica	5–15 anos (classificações funcionais não reportadas)	115	Consistência interna; Validade de critério; Teste de hipóteses.	**Consistência interna:** Muito bom **Validade de critério:** Muito bom **Teste de hipóteses:** Muito bom	**Consistência Interna**: α = 0.91 **Validade de Critério:** Área sob a curva: 0.720 **Teste de Hipóteses (Validade concorrente):** Fortes correlações entre as pontuações do DCDQ e as pontuações do M‐ABC (Spearman = 0,60) **Teste de Hipóteses (Validade Discriminante):** Correlações significativas entre o grupo ASD + DCD versus o grupo ASD sem DCD. ‐ Total do DCDQ: p < 0,001 ‐ Controle durante o movimento: p < 0,001 ‐ Motricidade fina/Escrita: p < 0,001 ‐ Coordenação geral: p < 0,001	**Consistência Interna:** Suficiente (+) **Validade de Critério:** Suficiente (+) **Teste de Hipóteses (Validade concorrente e discriminante):** Suficiente (+)
*MABC‐2 Transcultural Adaptation and Evaluation of Children Aged 7 to 10 Years With Autistic Spectrum Disorder* ^36^	Bateria de Avaliação do Movimento para Crianças‐2 (MABC‐2)	Brasil	7–10 anos (classificações funcionais não reportadas)	41	Adaptação Transcultural; Teste de hipóteses.	**Validade transcultural:** Inadequada **Teste de hipóteses:** Inadequada	**Adaptação Transcultural:** Não reportado **Teste de Hipóteses (Validade Convergente):** Correlações positivas com o teste de Matrizes Progressivas Coloridas de Raven (r = 0,17‐0,56, p < 0,05)	**Adaptação Transcultural:** Indeterminado (?) **Teste de Hipóteses (Validade Convergente):** Insuficiente (−)

Legenda: **ACSF‐SC**: Sistema de Classificação de Funcionalidade no Autismo ‐ Comunicação Social; **α**: alfa, TEA: Transtorno do Espectro Autista, **CFI:** Índice de Comparação de Ajuste, **MMD:** Mudança Mínima Detectável, **ICC:** Coeficiente de Correlação Intraclasse.

A Tabela 2 resume cada avaliação de risco de viés para cada propriedade de medida avaliada nos estudos selecionados. A pontuação completa do risco de viés, com os critérios que reduziram a pontuação final do risco de viés de cada estudo e de cada propriedade de medida, é apresentada no Material Suplementar 3.

### Qualidade das propriedades de medida e síntese dos resultados

Cada índice de propriedade de medida identificado nos estudos selecionados foi classificado em três categorias: “suficiente” (+), “insuficiente” (−) ou “indeterminado” (?). Aproximadamente 69,56% das propriedades de medida apresentaram resultados suficientes (+), 21,73% apresentaram resultados indeterminados (?) e o restante (8,71%) apresentou resultados insuficientes (−). Dentre os resultados suficientes: 16,6% eram resultados de confiabilidade, 33,3% referiam‐se a testes de hipóteses, 12% eram sobre consistência interna e 4% sobre validade de critério. Para os resultados indeterminados (?): 8% eram sobre erro de medida; 4% sobre consistência interna e 4% sobre adaptação transcultural, e os outros 4% sobre validade de conteúdo. Entre os resultados insuficientes (−): 4% estavam relacionados ao desenvolvimento do instrumento e aos testes de hipóteses. Em relação à validade estrutural, os resultados foram suficientes (+).

### Nível de evidência

**Figura 1:**
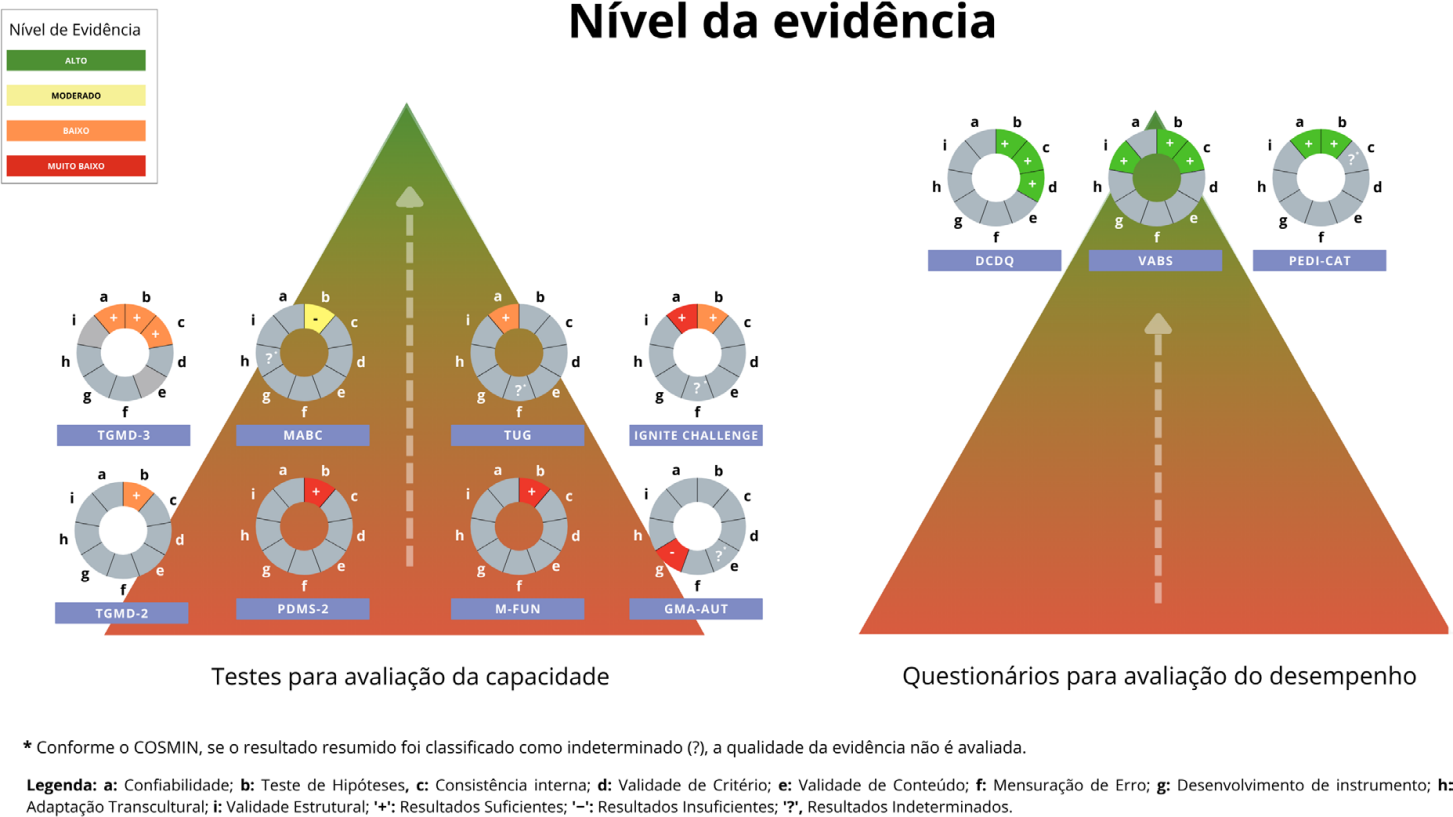
Representação do nível de evidência com base nas propriedades psicométricas. Nível de evidência GRADE: verde, alto; amarelo, moderado; laranja, baixo; vermelho, muito baixo. a, confiabilidade; b, teste de hipóteses; c, consistência interna; d, validade de critério; e, validade de conteúdo; f, mensuração de erro; g, desenvolvimento do instrumento; h: adaptação transcultural e i: validade estrutural ‘+’, resultados suficientes; ‘−’, resultados insuficientes; ‘?’, resultado indeterminado. DCDQ: Questionário de Transtorno do Desenvolvimento da Coordenação; GMA‐AUT: Avaliação Motora Grossa de Crianças e Adolescentes com Transtorno do Espectro Autista; MABC‐2: Bateria de Avaliação do Movimento para Crianças‐2; M‐FUN: Escalas de Função e Participação de Miller; PDMS‐2: Escalas de Desenvolvimento Motor de Peabody, Segunda Edição; PEDI‐CAT: Inventário de Avaliação Pediátrica de Incapacidade ‐ Teste adaptativo por computador; TGMD‐2,3: Teste de Desenvolvimento Motor Grosso ‐ segunda e terceira edições; TUG: Timed Up and Go; VABS: Escalas de Comportamento Adaptativo de Vineland.

Em relação ao nível de evidência, observou‐se que 39,13% das propriedades avaliadas foram classificadas como possuindo um nível de evidência “alto”, 4,34% como moderado, 34,78% como “baixo” e os restantes 21,73% como “muito baixo”. A Figura 1 fornece uma representação do nível de evidência conforme o número de propriedades avaliadas, a classificação GRADE e a qualidade das propriedades de medida. A figura foi elaborada considerando que sua base representa o nível de evidência mais baixo e índices de propriedades de medida insuficientes (−), enquanto o topo mostra os instrumentos com o mais alto nível de evidência e propriedades de medida suficientes (+). Dessa forma, os instrumentos com o maior nível de evidência e índices psicométricos apropriados para as propriedades avaliadas estão representados no topo. As classificações detalhadas do nível de evidência, seguindo os critérios GRADE, são fornecidas no Material Suplementar 4.

#### Confiabilidade

Dentre os estudos incluídos, cinco investigaram a confiabilidade de quatro instrumentos: Ignite Challenge^29^, PEDI‐CAT^30^, TGMD‐3^33^ e TUG^34^. TGMD‐3 e TUG foram classificados como possuindo um nível de evidência baixo devido à imprecisão muito séria na avaliação do TGMD‐3 e ao risco de viés muito sério associado à imprecisão séria no TUG. Ignite Challenge foi classificado como possuindo um nível de evidência muito baixo devido ao risco de viés sério e à imprecisão séria. PEDI‐CAT, no entanto, foi classificado como possuindo um nível de evidência alto, por não apresentar risco de viés e por demonstrar resultados consistentes.

#### Teste de Hipóteses

Nove instrumentos tiveram o teste de hipóteses avaliado como uma propriedade de medida: VABS^26^, ^27^, Ignite Challenge^29^, PEDI‐CAT^30^, M‐FUN e PDMS‐2^31^, TGMD‐2^32^, TGMD‐3^33^, DCDQ^35^ e M‐ABC‐2^36^. TGMD‐2, TGMD‐3 e Ignite Challenge foram classificados como possuindo um nível de evidência baixo devido à imprecisão muito séria. M‐FUN e PDMS‐2 foram classificados como possuindo um nível de evidência muito baixo devido ao risco de viés sério e à imprecisão muito séria. DCDQ, VABS e PEDI‐CAT foram classificados como possuindo um nível de evidência alto, apoiados por resultados consistentes. M‐ABC‐2 foi classificado como possuindo um nível de evidência baixo devido à imprecisão séria e ao risco de viés.

#### Consistência Interna

A consistência interna do VABS^26^, TGMD‐3^33^ e DCDQ^35^ foi avaliada. Um alto nível de evidência foi atribuído ao DCDQ e ao VABS devido a resultados consistentes e à ausência de risco de viés. TGMD‐3, no entanto, foi classificado como possuindo um nível de evidência baixo devido à imprecisão muito séria. PEDI‐CAT^30^ apresentou consistência interna indeterminada (?) e, portanto, nenhum nível de evidência pôde ser atribuído até o momento.

#### Validade de Critério

DCDQ^35^ foi classificado como possuindo um alto nível de evidência devido à ausência de risco de viés e a resultados consistentes.

#### Validade de Conteúdo

GMA‐AUT^28^ apresentou validade de conteúdo indeterminada (?), portanto, nenhum nível de evidência foi classificado.

#### Mensuração de Erro

Dois instrumentos, o Ignite Challenge^29^ e o TUG^34^, tiveram seu erro de medida avaliado. Ambos não tiveram nível de evidência classificado devido à qualidade indeterminada (?) da propriedade de medida.

#### Desenvolvimento do Instrumento

O GMA‐AUT^28^ foi classificado como possuindo um nível de evidência muito baixo devido a um risco de viés extremamente sério e imprecisão séria.

#### Adaptação Transcultural

M‐ABC‐2^36^ foi classificado como possuindo um nível de evidência baixo devido à imprecisão séria e ao risco de viés.

#### Validade Estrutural

VABS^26^ foi classificado como possuindo um alto nível de evidência devido à ausência de risco de viés e a resultados consistentes.

### Mapa de decisão clínica baseado no nível de evidência

**Figura 2:**
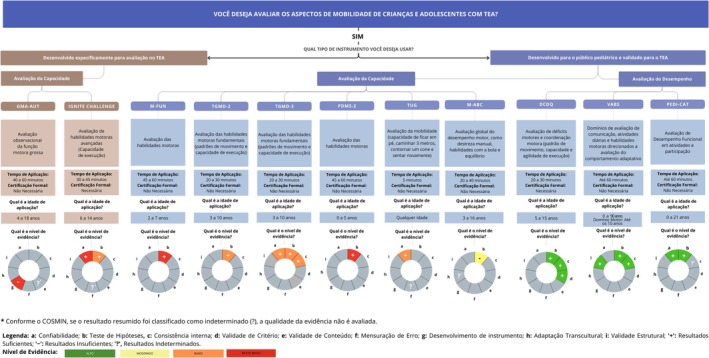
Mapa de decisão clínica. a, confiabilidade; b, teste de hipóteses; c, consistência interna; d, validade de critério; e, validade de conteúdo; f, mensuração de erro; g, desenvolvimento do instrumento; h: adaptação transcultural e i: validade estrutural ‘+’, resultados suficientes; ‘−’, resultados insuficientes; ‘?’, resultado indeterminado. DCDQ: Questionário de Transtorno do Desenvolvimento da Coordenação; GMA‐AUT: Avaliação Motora Grossa de Crianças e Adolescentes com Transtorno do Espectro Autista; MABC‐2: Bateria de Avaliação do Movimento para Crianças‐2; M‐FUN: Escalas de Função e Participação de Miller; PDMS‐2: Escalas de Desenvolvimento Motor de Peabody, Segunda Edição; PEDI‐CAT: Inventário de Avaliação Pediátrica de Incapacidade ‐ Teste adaptativo por computador; TGMD‐2,3: Teste de Desenvolvimento Motor Grosso ‐ segunda e terceira edições; TUG: Timed Up and Go; VABS: Escalas de Comportamento Adaptativo de Vineland.

O mapa de decisão clínica resume os dados encontrados nesta revisão, apontando os aspectos variáveis incluídos nos itens dos instrumentos e a faixa etária abrangida por cada um deles na Figura 2.

## DISCUSSÃO

Esta revisão sistemática teve como objetivo investigar os instrumentos disponíveis para avaliar aspectos da mobilidade e analisar a qualidade e o nível de evidência de suas propriedades de medida na população com TEA. Foram identificados nove instrumentos desenvolvidos para crianças com desenvolvimento típico e validados para o TEA, juntamente com dois instrumentos desenvolvidos especificamente para o TEA. Evidências positivas e altas foram encontradas para o DCDQ (teste de hipóteses, consistência interna e validade de critério), VABS (teste de hipóteses, consistência interna e validade estrutural) e PEDI‐CAT (confiabilidade e teste de hipóteses). Por outro lado, evidências positivas baixas foram encontradas para o Ignite Challenge (teste de hipóteses), TGMD‐2 (teste de hipóteses), TGMD‐3 (confiabilidade, teste de hipóteses e consistência interna), TUG (confiabilidade) e M‐ABC (teste de hipóteses e adaptação transcultural). Evidências positivas muito baixas foram identificadas para o Ignite Challenge (confiabilidade), PDMS‐2 (teste de hipóteses) e M‐FUN (teste de hipóteses). O GMA‐AUT apresentou evidência negativa, baixa e indeterminada para validade de conteúdo e desenvolvimento do instrumento. Os resultados mostram que há mais evidências altas em relação aos instrumentos de desempenho em comparação com os de capacidade.

As classificações neste estudo revelam a qualidade e o nível de evidência de instrumentos projetados para avaliar aspectos funcionais da mobilidade. Estes instrumentos avaliam diferentes construtos dos aspectos de mobilidade dentro dos conceitos de desempenho e capacidade. Vale ressaltar que nenhum dos estudos avaliou todas as propriedades de medida consideradas importantes pelo COSMIN.^23^ Cada instrumento revisado apresenta vantagens e limitações distintas, particularmente no que diz respeito à abrangência das propriedades avaliadas e sua aplicabilidade na prática clínica. Com base nos achados desta revisão, o DCDQ, VABS e PEDI‐CAT são os instrumentos com o mais alto nível de evidência para avaliar aspectos de mobilidade em crianças e adolescentes com TEA. Adicionalmente, VABS e PEDI‐CAT destacam‐se por sua abrangência na avaliação de outros aspectos relevantes da funcionalidade, como atividades diárias, comunicação e responsabilidades sociais.^26^, ^27^, ^30^ Os resultados para estes instrumentos foram geralmente consistentes e suficientes para investigar confiabilidade, teste de hipóteses, validade de critério e validade estrutural, garantindo seu uso na prática clínica.

Os instrumentos de capacidade GMA‐AUT, Ignite Challenge, PDMS‐2, M‐FUN, TGMD‐2, TGMD‐3, TUG e M‐ABC apresentaram níveis de evidência de moderado a muito baixo para suas propriedades avaliadas. A imprecisão e o risco de viés foram fatores‐chave que afetaram sua evidência. O risco de viés foi determinado de acordo com os critérios de avaliação para cada propriedade avaliada.^23^ A imprecisão foi baseada no tamanho da amostra incluído em cada estudo.^25^ Os tamanhos de amostra para crianças e adolescentes com TEA foram notavelmente menores para os instrumentos de capacidade em comparação com os questionários de desempenho. Isto pode refletir a relativa facilidade de administrar questionários padronizados para os pais versus a realização de avaliações observacionais presenciais necessárias para os testes de capacidade. Consequentemente, os questionários de desempenho tendem a ter níveis de evidência mais altos do que os testes de capacidade em crianças com TEA. Estudos futuros devem priorizar a avaliação de instrumentos de capacidade com tamanhos de amostra adequados para reduzir a imprecisão.

Instrumentos como o VABS, PEDI‐CAT, PDMS‐2, TGMD e M‐ABC são amplamente estudados em diferentes transtornos do neurodesenvolvimento. Até onde sabemos, não há nenhuma revisão sistemática que tenha avaliado a qualidade de suas propriedades psicométricas, seguindo os padrões COSMIN, considerando a população geral com deficiências. No entanto, reconhecemos que estes instrumentos podem demonstrar diferentes níveis de evidência quanto à qualidade de suas propriedades de medida ao considerar populações com transtornos do neurodesenvolvimento de forma mais ampla. Para fornecer interpretações mais específicas do nível de evidência para seu uso em crianças e adolescentes com TEA, são necessários estudos futuros com tamanhos de amostra maiores e qualidade metodológica mais robusta para fortalecer a base de evidências para estes instrumentos.

Além disso, a investigação das propriedades do Ignite Challenge e do M‐FUN carece de estudos que avaliem melhor sua aplicabilidade e validade. Até à data, poucos estudos se dedicaram a esta investigação. Nos resultados do estudo de Wright et al. (2022), as propriedades de confiabilidade e teste de hipóteses avaliadas mostraram resultados suficientes.^29^ Da mesma forma, no estudo de Wright, o teste de hipóteses avaliado por Holloway et al. (2018) também produziu resultados consistentes.^31^ Possivelmente, com uma investigação futura mais aprofundada, o nível de evidência para estes instrumentos pode mudar. Estudos metodológicos futuros focados na investigação de instrumentos de avaliação para TEA devem garantir que os aspectos metodológicos específicos relacionados às propriedades examinadas e o tamanho da amostra sejam adequados e tenham poder estatístico suficiente para sustentar a qualidade da evidência investigada.

A confiabilidade é uma propriedade importante a considerar.^24^, ^37^ Dos resultados, apenas 5 dos 11 instrumentos avaliaram esta propriedade de medida. A confiabilidade refere‐se ao grau em que os pacientes podem ser diferenciados uns dos outros, considerando os erros de medida.^37^ Por exemplo, nenhum estudo investigou a confiabilidade do TGMD‐2 em amostras de crianças com TEA, apenas comparando uma pequena porção de crianças com deficiências com crianças com desenvolvimento típico. Dada a variabilidade dos comprometimentos motores no TEA, é evidente a importância de um estudo que investigue especificamente estes dados normativos para o TEA. Estes achados estão alinhados com os dados apresentados pela revisão de Wilson et al. (2018), que mostra que a maior limitação estava relacionada à ausência de crianças e adolescentes com TEA nas amostras normativas dos instrumentos.^17^

Assim como a confiabilidade, a responsividade é uma propriedade que deve ser investigada. De acordo com Terwee et al. (2007), a responsividade define a capacidade de um instrumento de detectar mudanças clinicamente importantes ao longo do tempo, mesmo que pequenas.^37^ Nenhum dos 11 estudos incluídos avaliou a responsividade dos instrumentos. Avaliar a responsividade é fundamental, pois determina a capacidade do instrumento de detectar mudanças significativas ao longo do tempo, especialmente em contextos de intervenção clínica.^22^, ^37^ Para crianças e adolescentes com TEA, esta análise é particularmente relevante, pois ajuda a identificar se as mudanças observadas refletem efeitos tangíveis de uma intervenção. Estudos que avaliam a responsividade contribuem para o avanço científico, garantindo que os instrumentos não são apenas válidos e confiáveis, mas também sensíveis a mudanças críticas para a funcionalidade dos indivíduos com TEA.

Outra propriedade importante, investigada somente em um estudo incluído, é a adaptação transcultural. Esta propriedade diz respeito à medida em que os itens de um instrumento traduzido ou adaptado culturalmente refletem adequadamente os itens do instrumento original.24 A adaptação transcultural garante que as propriedades psicométricas do instrumento original sejam preservadas na versão adaptada, assegurando que ele continue a medir os construtos pretendidos em diferentes contextos culturais. Alguns dos instrumentos incluídos, como o PEDI‐CAT e o TGMD2, são amplamente traduzidos transculturalmente; no entanto, como o processo de tradução transcultural não incluiu indivíduos com TEA, esses estudos não foram incluídos nesta revisão.^38^, ^39^

É importante destacar que a avaliação dos aspectos de mobilidade em crianças com TEA pode ser complementada usando outras avaliações de Funções e Estruturas do Corpo. Após avaliar as limitações de desempenho e capacidade em crianças e adolescentes com TEA, os terapeutas devem investigar quais componentes motores (por exemplo, equilíbrio, coordenação, força) podem estar dificultando a mobilidade das crianças. Para isso, o uso de instrumentos populares, como o Teste de Proficiência Motora de Bruininks‐Oseretsky (BOT), pode ser útil.^40^, ^41^

### Limitações e Direções Futuras

O critério de elegibilidade exigiu deliberadamente a inclusão de estudos que investigaram propriedades de medida em amostras predominantemente compostas por crianças e adolescentes com TEA, para garantir que as propriedades psicométricas analisadas fossem especificamente direcionadas a esta população. Esta decisão foi essencial para preservar a relevância clínica e a validade dos achados, uma vez que a inclusão de estudos metodológicos nos quais menos de 50% dos participantes tinham TEA poderia levar a uma indireção séria e comprometer a precisão do nível de evidência. Ao manter este foco, a revisão fornece informações baseadas em evidências representativas da população com TEA, o que suporta uma tomada de decisão clínica mais precisa e adequada ao contexto.

Reconhece‐se, no entanto, que outras ferramentas desenvolvidas para populações mais amplas com transtornos do neurodesenvolvimento ainda podem ser alternativas úteis quando os instrumentos aqui incluídos não atendem plenamente aos objetivos do terapeuta. Nesses casos, a seleção da ferramenta de avaliação mais adequada deve ser sempre guiada pelas necessidades e objetivos específicos da criança e da família, bem como pelas características individuais da criança que está sendo avaliada.

A variabilidade no conteúdo dos instrumentos (ou seja, a inclusão de diferentes aspectos da mobilidade) e as diversas faixas etárias dos participantes dificultaram comparações mais aprofundadas para além de capacidade e desempenho. A escolha de um instrumento deve considerar primordialmente as características, os objetivos e o contexto do indivíduo. O mapa de decisão clínica apresentado neste estudo pode auxiliar os clínicos na seleção dos instrumentos de mobilidade mais apropriados para diferentes situações.

Considerando os desafios da avaliação de crianças com TEA, especialmente em testes de capacidade, estudos futuros devem incluir amostras maiores de crianças e adolescentes com TEA para estabelecer níveis de evidência específicos para esta população. Ademais, a falta de informação nos estudos incluídos sobre as classificações funcionais do TEA e os métodos de diagnóstico limitou a possibilidade de conduzir uma análise mais profunda e matizada dos resultados. Adicionalmente, pesquisas futuras deveriam investigar propriedades que ainda não foram avaliadas, como a responsividade.

## Conclusões

Esta revisão investigou a qualidade e o nível de evidência das propriedades de medida de nove instrumentos utilizados para avaliar os aspectos funcionais da mobilidade. A maioria das propriedades de medida dos instrumentos demonstrou evidência baixa e/ou muito baixa devido ao risco de viés e à imprecisão. DCDQ, VABS e PEDI‐CAT foram identificados como os instrumentos com o mais alto nível de recomendação quanto à sua evidência para avaliar aspectos de mobilidade em crianças e adolescentes com TEA. Em contraste, o Ignite Challenge, PDMS‐2, M‐FUN, GMA‐AUT, TGMD‐2, TGMD‐3, TUG e M‐ABC apresentaram níveis de evidência de moderado a muito baixo para as propriedades avaliadas. Os achados destacaram a necessidade de análises futuras das propriedades de medida dos instrumentos para avaliação de aspectos da mobilidade, seguindo as diretrizes do COSMIN.

### AGRADECIMENTOS

Este estudo foi financiado pela Coordenação de Aperfeiçoamento de Pessoal de Nível Superior (CAPES) por meio de bolsas de doutorado e de pesquisa.

## Supporting information


**Figura S1:** Fluxograma.

**Tabela S1:** Estratégia de busca conduzida em outubro de 2023.

**Tabela S2:** Resultados resumidos de acordo com o Checklist de Risco de Viés COSMIN.

**Table S3:** Summarized results according to the measurement properties
