# Supplementary material for: Instrumentos que avaliam a mobilidade de crianças e adolescentes com transtorno do espectro autista: Uma revisão sistemática e mapa de decisão
Source: Dev Med Child Neurol. 2025 Dec 29;68(8):e118–32. doi: 10.1111/dmcn.70144 (PMC13340618; doi:10.1111/dmcn.70144)
Supplement: Supplementary file 1 — Figura S1: Fluxograma. [file DMCN-68-e118-s003.docx]

Busca Manual (n= 10)

Amostras não compostas por indivíduos com TEA (9);

Não avaliaram o desfecho de mobilidade (2);

Resumos (1);

Instrumentos de Triagem (2).

**Seleção**


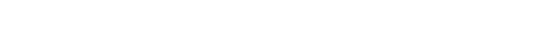


**Eligibilidade**

Registros identificados por meio da busca nos bancos de dados:

(n = 6098)

Duplicatas removidas

(n = 1397)

Registros analisados

(

n

=4711)

Resumos excluídos após leitura

=4686)

n

(

Artigos em texto completo avaliados quanto à elegibilidade (n=25)

Artigos de texto completo excluídos: (n=14)

Estudos Incluídos

(n = 11)

**Incluídos**


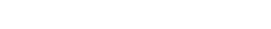


**Identificação**


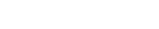


Figura S1. Fluxograma
