# Supplementary material for: Instrumentos que avaliam a mobilidade de crianças e adolescentes com transtorno do espectro autista: Uma revisão sistemática e mapa de decisão
Source: Dev Med Child Neurol. 2025 Dec 29;68(8):e118–32. doi: 10.1111/dmcn.70144 (PMC13340618; doi:10.1111/dmcn.70144)
Supplement: Supplementary file 3 — Tabela S2: Resultados resumidos de acordo com o Checklist de Risco de Viés COSMIN. [file DMCN-68-e118-s001.docx]

**Table S2**: Resultados resumidos de acordo com o Checklist de Risco de Viés COSMIN.

| **Instrumento** | **Propriedades de medida** | **Pontuação do Risco de Viés** | **Critérios que reduziram a pontuação final de risco de viés** |
| --- | --- | --- | --- |
| **Avaliação Motora Grossa de Crianças e Adolescentes com Transtorno do Espectro Autista (GMA-AUT)**  Heidrich et al., 2018 | Validade de Conteúdo | Inadequado | Apenas métodos quantitativos (de pesquisa) foram utilizados ou assumido que o método era apropriado, mas não descrito claramente, e assumido que a abordagem era apropriada, mas não descrita claramente |
|  | Desenvolvimento de Instrumento | Inadequado | Método utilizado não é apropriado ou adequado para o construto ou população do estudo |
| **Ignite Challenge**  Wright et al., 2023 | Confiabilidade | Adequado | Assumiu-se que os pacientes estavam estáveis |
|  | Teste de Hipóteses | Muito bom | Não se aplica |
|  | Mensuração de Erro | Adequado | Assumiu-se que os pacientes estavam estáveis |
| **Escalas de Função e Participação de Miller (M-FUN) e Escalas de Desenvolvimento Motor de Peabody, Segunda Edição (PDMS-2)**  Holloway et al., 2019 | Teste de Hipóteses | Adequado | Propriedades de medição suficientes do(s) instrumento(s) comparador(es), mas não se sabe se se aplicam à população do estudo |
| **Teste de Desenvolvimento Motor Grosso - Segunda edição (TGMD-2)**  Breslin et al., 2011 | Teste de Hipóteses | Muito bom | Não se aplica |
| **Teste de Desenvolvimento Motor Grosso - Terceira edição (TGMD-3)**  Allen et al., 2017 | Confiabilidade | Muito bom | Não se aplica |
|  | Teste de Hipóteses | Muito bom | Não se aplica |
|  | Consistência Interna | Muito bom | Não se aplica |
| **Escalas de Desenvolvimento Motor de Peabody, Segunda Edição (PDMS-2)**  Holloway et al., 2019 | Teste de Hipóteses | Adequado | Propriedades de medição suficientes do(s) instrumento(s) comparador(es), mas não se sabe se se aplicam à população do estudo |
| **Timed Up and Go (TUG)**  Martin-Diaz et al., 2023 | Confiabilidade | Adequado | Assumiu-se que os pacientes estavam estáveis |
|  | Mensuração de Erro | Adequado | Assumiu-se que os pacientes estavam estáveis |
| **Bateria de Avaliação do Movimento para Crianças-2 (MABC-2)**  Quedas et al., 2021 | Teste de Hipóteses | Inadequado | Os construtos medidos pelo(s) instrumento(s) comparador(es) não estão claros |
|  | Cross-Cultural Adaptation | Inadequado | Métodos não apropriados |
| **Questionário de Transtorno do Desenvolvimento da Coordenação (DCDQ)**  Van Damme et al., 2022 | Teste de Hipóteses | Muito bom | Não se aplica |
|  | Consistência Interna | Muito bom | Não se aplica |
|  | Validade de Critério | Muito bom | Não se aplica |
| **Escalas de Comportamento Adaptativo de Vineland (VABS)**  Deng et al., 2025; Bhat et al., 2024 | Teste de Hipóteses | Muito bom | Não se aplica |
|  | Consistência Interna | Muito bom | Não se aplica |
|  | Validade Estrutural | Muito bom | Não se aplica |
|  | Teste de Hipóteses | Adequado | Supõe-se que o método estatístico era apropriado |
| **Inventário de Avaliação Pediátrica de Incapacidade - Teste adaptativo por computador (PEDI-CAT)**  Chamberlain et al., 2024; Wright et al.,2023 | Confiabilidade | Adequado | Assumiu-se que os pacientes estavam estáveis |
|  | Teste de Hipóteses | Muito bom | Não se aplica |
|  | Consistência Interna | Muito bom | Não se aplica |
