# Supplementary material for: Instrumentos que avaliam a mobilidade de crianças e adolescentes com transtorno do espectro autista: Uma revisão sistemática e mapa de decisão
Source: Dev Med Child Neurol. 2025 Dec 29;68(8):e118–32. doi: 10.1111/dmcn.70144 (PMC13340618; doi:10.1111/dmcn.70144)
Supplement: Supplementary file 4 — Table S3: Summarized results according to the measurement properties [file DMCN-68-e118-s002.docx]

**Table S3:** Resultados resumidos de acordo com as propriedades de medidas.

| **Confiabilidade** | **Resultados resumidos** | **Amostra Total** | **Avaliação Geral** | **Nível de Evidência (GRADE)** |
| --- | --- | --- | --- | --- |
| **Teste de Desenvolvimento Motor Grosso (TGMD-3)**  Allen et al., (2017) | ICC= variando entre 0.91-0.99, | n=14 | Suficiente (+) | **Baixo nível de evidência** (imprecisão muito séria) |
| **Timed Up and Go (TUG)**  Martín-Díaz et al., (2023) | ICC= variando entre 0.88-0.99 | n= 50 | Suficiente (+) | **Baixo nível de evidência** (risco muito sério de viés e imprecisão grave) |
| **Ignite Challenge**  Wright et al., 2023 | ICC= variando entre 0.91-0.96 | n=47 | Suficiente (+) | **Muito baixo nível de evidência** (risco sério de viés e imprecisão muito grave) |
| **Inventário de Avaliação Pediátrica de Incapacidade - Teste adaptativo por computador (PEDI-CAT)**  Chamberlain et al., 2024 | ICC= variando entre 0.89-0.92 | n=134 | Suficiente (+) | **Alto nível de Evidência** (sem risco de viés e resultados consistentes) |

| **Teste de Hipóteses** | | **Resultados resumidos** | **Amostra Total** | **Avaliação Geral** | **Nível de Evidência (GRADE)** |
| --- | --- | --- | --- | --- | --- |
| **Teste de Desenvolvimento Motor Grosso (TGMD-2)**  Breslin et al., 2011 | | Diferença significativa entre os grupos com e sem suporte visual p=0,003 | n=22 | Suficiente (+) | **Baixo nível de evidência** (imprecisão muito séria) |
| **Teste de Desenvolvimento Motor Grosso (TGMD-3)**  Allen et al., 2017 | | Os escores brutos do TGMD-3 de crianças com TEA melhoraram significativamente com o uso do suporte visual do TGMD-3 (p=0,01). Melhoria significativa com o uso de suportes visuais (p=0,01). | n=15 | Suficiente (+) | **Baixo nível de evidência** (imprecisão muito séria) |
| **Escalas de Função e Participação de Miller (M-FUN)**  Holloway et al., 2019 | | Validade concorrente: Correlação significativa entre a escala M-FUN e os escores de motricidade grossa do PDMS-2 (r= 0,84, p<0,05).  Validade discriminante: Forte correlação na identificação de crianças com habilidades motoras médias e atrasadas (k de Cohen = 0,77 e p < 0,05). | n=22 | Suficiente (+) | **Muito baixo nível de Evidência** (risco sério de viés e imprecisão muito grave) |
| **Escalas de Desenvolvimento Motor de Peabody, Segunda Edição (PDMS-2)**  Holloway et al., 2019 | |  |  |  |  |
| **Ignite Challenge**  Wright et al., 2023 | | Validade concorrente: Correlações significativas entre os escores do Ignite Challenge e o domínio de mobilidade do PEDI-CAT (r= 0,54, p <0,0001) e Social/Cognitivo (r = 0,57, p<0,0001)  Validade discriminante: O Ignite Challenge mostra resultados diferentes entre faixas etárias (melhor escore em crianças mais jovens 59,4 (dp=15,5) versus escore em crianças mais velhas 80,3 (dp=10,1), p < 0,001), e também entre níveis ACSF:SC (Nível I melhor escore 73,8 (dp=12,8) versus nível II escore 58,3 (dp=20,9), p= 0,007). | n= 47 | Suficiente (+) | **Baixo nível de evidência** (imprecisão muito séria) |
| **Questionário de Transtorno do Desenvolvimento da Coordenação (DCDQ)**  Van Damme et al., 2022 | | Validade concorrente:  Forte correlação entre os escores do DCDQ e os escores do M-ABC (Spearman = 0,60)  Validade discriminante: Correlações significativas entre o grupo ASD + DCD versus o grupo ASD sem DCD.  Total do DCDQ: p<0,001  - Controle durante o movimento: p<0,001  - Motricidade fina/Escrita: p<0,001  - Coordenação geral: p<0,001 | n=115 | Suficiente (+) | **Alto nível de Evidência** (sem risco de viés e resultados consistentes) |
| **Inventário de Avaliação Pediátrica de Incapacidade - Teste adaptativo por computador (PEDI-CAT)**  Chamberlain et al., 2024 | | Validade convergente:  Correlações positivas significativas VABS (r=0,51-0,74, p<0,05) | N=134 | Suficiente (+) | **Alto nível de Evidência** (sem risco de viés e resultados consistentes) |
| **Bateria de Avaliação do Movimento para Crianças-2 (MABC-2)**  Quedas et al., 2021  Van Damme et al., 2022 | | Validade convergente:  Correlações positivas e significativas com os Cronogramas de Desenvolvimento de Gesell (r=0,30-0,60, p<0,05) | n=156 | Insuficiente (-) | **Moderado nível de evidência** (risco sério de viés) |
| **Escalas de Comportamento Adaptativo de Vineland (VABS)** | Deng et al., 2025 | Validade concorrente: Observou-se correlação significativa entre o CVABS-III e os Cronogramas de Desenvolvimento de Gesell (GDS), particularmente no grupo de 3 a 6 anos, com fortes associações nos domínios de comunicação (r=0,79, r=0,79), habilidades da vida diária (r=0,44–0,53, r=0,44–0,53) e socialização (r=0,47–0,53, r=0,47–0,53), todos p<0,001, p<0,001.  Validade discriminante: O CVABS-III diferenciou efetivamente entre crianças com desenvolvimento típico e aquelas com TEA ou DD em todas as faixas etárias, com grandes tamanhos de efeito (d de Cohen >0,7 em múltiplas áreas para idades de 3 a 6 anos, p<0,001), mostrando diferenças significativas, porém menores, para crianças com SLDD. | n=2713 | Suficiente (+) | **Alto nível de Evidência** (sem risco de viés e resultados consistentes) |
|  | Bhat et al., 2024 | Validade concorrente: Correlação significativa entre o DCD-Q e os escores do domínio motor das Escalas de Comportamento Adaptativo de Vineland (VABS) (r=0,62, p<0,0001).  Validade discriminante: Alta precisão (81,2%) na identificação de atrasos motores concorrentes entre o DCD-Q e o VABS, com valor preditivo positivo de 70,4%. |  |  |  |

| **Consistência Interna** | **Resultados resumidos** | **Amostra Total** | **Avaliação Geral** | **Nível de Evidência (GRADE)** |
| --- | --- | --- | --- | --- |
| **Teste de Desenvolvimento Motor Grosso (TGMD-3)**  Allen et al., 2017 | Com suporte visual: alfa de Cronbach de 0,93;  Sem suporte visual: alfa de Cronbach de 0,88; | n=14. | Suficiente (+) | **Baixo nível de evidência** (imprecisão muito séria) |
| **Questionário de Transtorno do Desenvolvimento da Coordenação (DCDQ)**  Van Damme et al., 2022 | Alfa de Cronbach de 0.91; | n=115 | Suficiente (+) | **Alto nível de Evidência** (sem risco de viés e resultados consistentes) |
| **Inventário de Avaliação Pediátrica de Incapacidade - Teste adaptativo por computador (PEDI-CAT)**  Chamberlain et al., 2024 | Omega de McDonald entre 0.89 - 0.93; | N=134 | Indeterminado (?) | **Alto nível de Evidência** (sem risco de viés e resultados consistentes) |
| **Escalas de Comportamento Adaptativo de Vineland (VABS)**  Deng et al., 2025 | Alfa de Cronbach de 0.93-0.99 | n=2252 | Suficiente (+) | **Alto nível de Evidência** (sem risco de viés e resultados consistentes) |

| **Validade de Critério** | **Resultados resumidos** | **Amostra Total** | **Avaliação Geral** | **Nível de Evidência (GRADE)** |
| --- | --- | --- | --- | --- |
| **Questionário de Transtorno do Desenvolvimento da Coordenação (DCDQ)**  Van Damme et al., 2022 | Area sob a curva 0.72 | n=115 | Suficiente (+) | **Alto nível de Evidência** (sem risco de viés e resultados consistentes) |

| **Validade de Conteúdo** | **Resultados resumidos** | **Amostra Total** | **Avaliação Geral** | **Nível de Evidência (GRADE)** |
| --- | --- | --- | --- | --- |
| **Avaliação Motora Grossa de Crianças e Adolescentes com Transtorno do Espectro Autista (GMA-AUT)**  Heidrich et al., 2022 | IVC* entre 0.88 a 1.00  *Indice de Validade de Conteúdo (IVC) | n= 8 | Indeterminado (?) | **Muito baixo nível de evidência** (risco de viés extremamente sério) |

| **Mensuração de Erro** | **Resultados resumidos** | **Amostra Total** | **Avaliação Geral** | **Nível de Evidência (GRADE)** |
| --- | --- | --- | --- | --- |
| **Timed Up and Go (TUG)**  Martin-Diaz et al., 2023 | Erro padrão de medição de 0,02; com mudança mínima detectável de 0,06. | n= 50 | Indeterminado (?) | **Baixo nível de evidência** (risco sério de viés e séria imprecisão) |
| **Ignite Challenge**  Wright et al., 2023 | Erro de medição padrão de 5,13; com mudança mínima detectável de 9,28. | n= 47 | Indeterminado (?) | **Muito baixo nível de evidência** (risco sério de viés e imprecisão muito grave) |

| **Desenvolvimento de Instrumento** | **Resultados resumidos** | **Amostra Total** | **Avaliação Geral** | **Nível de Evidência (GRADE)** |
| --- | --- | --- | --- | --- |
| **Avaliação Motora Grossa de Crianças e Adolescentes com Transtorno do Espectro Autista (GMA-AUT)**  Heidrich et al., 2022 | Na versão final do instrumento, apenas dois itens apresentaram IVC* de 0,88, enquanto todos os outros tiveram IVC* de 1,00.  *Índice de Validade de Conteúdo (IVC) | n= 8 | Insuficiente (-) | **Muito baixo nível de evidência** (risco de viés extremamente sério e imprecisão muito grave) |

| **Adaptação Transcultural** | **Resultados resumidos** | **Amostra Total** | **Avaliação Geral** | **Nível de Evidência (GRADE)** |
| --- | --- | --- | --- | --- |
| **Bateria de Avaliação do Movimento para Crianças-2 (MABC-2)**  Quedas et al., 2021 | Observou-se que as correlações com os escores padrão entre os domínios avaliados pelo MABC-2 e os resultados do CPM mostraram ser medianas para Destreza Manual (r = 0,454, p = 0,012) e Equilíbrio (r = 0,324, p = 0,081) e pequenas para Mirar e Pegar (r = 0,170, p = 0,368). | n=41 | Indeterminado (?) | **Baixo nível de evidência** (risco sério de viés e inconsistência séria) |

| **Validade Estrutural** | **Resultados resumidos** | **Amostra Total** | **Avaliação Geral** | **Nível de Evidência (GRADE)** |
| --- | --- | --- | --- | --- |
| **Escalas de Comportamento Adaptativo de Vineland (VABS)**  Deng et al., 2025 | CFI=0.90-0.99 | N=2252 | Suficiente (+) | **Alto nível de Evidência** (sem risco de viés e resultados consistentes) |
